# Improved Specificity and False Discovery Rates for Multiplex Analysis of Changes in Strain-Specific Anti-Influenza IgG

**DOI:** 10.1155/2019/3053869

**Published:** 2019-04-15

**Authors:** Dongmei Li, Jiong Wang, John J. Treanor, Martin S. Zand

**Affiliations:** ^1^Informatics Core, Clinical and Translational Science Institute, University of Rochester Medical Center, Rochester, NY, USA; ^2^Department of Medicine, Division of Nephrology, University of Rochester Medical Center, Rochester, NY, USA; ^3^Department of Medicine, Division of Infectious Diseases, University of Rochester Medical Center, Rochester, NY, USA; ^4^Rochester Center for Health Informatics, University of Rochester Medical Center, Rochester, NY, USA

## Abstract

We describe a statistical approach to compare absolute antibody concentrations, both within and across subjects, derived from a multidimensional measurement of IgG binding to the influenza surface receptor hemagglutinin (HA). This approach addresses a fundamental problem in the field of vaccine immunology: how to accurately compare the levels of antibodies against multiple influenza strains. The mPlex-Flu assay can simultaneously measure the concentration of IgG antibodies against up to 50 influenza strains with only ≤10  *μl* of serum. It yields mean fluorescence intensity (MFI) over a 4-log range with low inter- and intrasample variability. While comparison of IgG binding to a single HA between subjects is straightforward, variations in binding behavior across influenza strains, coupled with reagent variations, make quantifying and comparing binding between multiple HA subtypes within subjects challenging. In this paper, we first treat such HA variations as an independent antigen and calculate each subtype antibody concentration using its own standard curve, normalizing variations in HA binding. We applied this method to the analyses of data from an H5 influenza clinical vaccine study. The results demonstrated that there are differences in coefficient estimates and in results of “comparing groups” between those with versus those without consideration of subtype antibody variations. Then, we used simulation studies to show the importance of taking the subtype antibody variations into account in HA strain antibody data analysis. Using a common standard curve for all subtype antibodies resulted in both inflated type I error and lowered specificity when comparing different treatment groups. Our results suggest that using individual standard curves for each influenza HA strain, and independently calculating anti-HA IgG concentrations, allows for adjustment of influenza HA subtype variations in treatment group comparisons in clinical vaccine studies. This method facilitates the direct comparison of serum anti-HA IgG concentrations against different influenza HA subtypes for multiplex assays.

## 1. Introduction

Estimating the concentration of antibodies directed against the major influenza viral surface protein hemagglutinin (HA) is critical for studies of antibody-mediated influenza immunity and especially for vaccine development [1]. Because the influenza virus mutates frequently, new strains are always emerging that can evade prior anti-HA IgG-mediated immunity, necessitating new vaccine formulations each year. Recently, emphasis has been placed on creating vaccines that generate broadly cross-reactive antibodies, protecting against many influenza strains [2]. Thus, the ability to simultaneously measure antibody binding against multiple influenza HA and to accurately compare antibody binding across many influenza strains, especially within and between subject binding distributions, is highly desirable. However, a major impediment to such comparisons is the variability of such multiple comparisons across many HA reagents, both for technical and statistical reasons.

We have previously described a multiplex-based method that simultaneously measures antibody binding against up to 50 influenza strain hemagglutinin proteins, the mPlex-Flu assay [3, 4]. HA proteins mediate viral attachment and entry into target cells [5]. Antibodies that bind to influenza HA can prevent or attenuate the severity of influenza infection. In mPlex-flu assay, each recombinant influenza strain HA couples to fluorescent microbeads; then, the mixtures of the HA-coated beads are used to simultaneously detect antibodies binding to multiple influenza strains. This multidimensional analytic method generates a continuous value for the mean fluorescence intensity (MFI), accurate over a 4-log range, reflecting antibody binding.

As a multidimensional assay, mPlex-flu assay is different from traditional titer-based assays such as the hemagglutinin inhibition (HAI) [6, 7] and microneutralization (MN) [8, 9] assays that measure IgG antibody binding to single-HA proteins. Importantly, this feature allows for the measurement of multidimensional cross-reactive immunity [4, 10], which is crucial when assessing whether a vaccine will provide broad protection against many influenza strains. This assay provides accurate concentrations of anti-HA IgG against different influenza strains and is able to detect statistically significant variations between experimental groups in clinical vaccine studies, compared to the HAI and MN assays.

**Figure 1:**
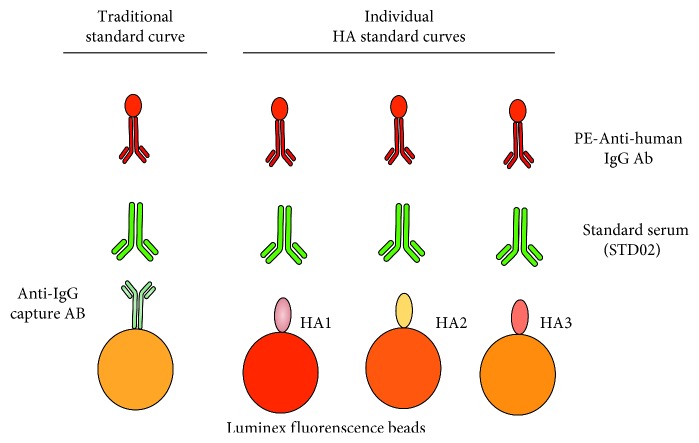
Principal of the mPLEX-Flu assay standard curve generation.

Translating MFI measured by multiplex assay into absolute concentrations of anti-HA antibodies creates unique challenges compared to standard monoplex semiquantitative assays (e.g., ELISA, HAI). First, mPlex-Flu assay uses influenza strain-specific rHA coupling microbeads to detect the anti-HA antibodies. However, traditional quantitative assays (e.g., ELISA [11], Luminex assay [12]) use immunoglobulin-specific capture antibody to couple microbeads to estimate the antibodies concentrations. Second, between-strain differences in HA molecular properties can cause slight variations in the density of the different HA's coating multiplex beads, resulting in slightly altered HA saturation and IgG binding characteristics [3, 4] (Figure 1). In addition, the assay is used to measure binding of a mixture of antibodies in sera that bind to multiple different sites on the HA protein, each with different affinities. The assessed antibody responses are polyclonal, but creating a precise mixture of monoclonal antibodies, targeting 20–40 different subtypes of influenza HA protein for 40–50 different HA, is technically unfeasible. Thus, a mixture of polyclonal sera with reactivities against all HA strains must be used. Finally, traditional statistical methods for analyzing concentration data [11, 13], using one common standard curve for all subtypic HAs [12], do not account for reagent binding differences between captured proteins. This may lead to increased Type I statistical errors and lowered specificity, when comparing treatment groups in clinical vaccine studies.

To address these issues, to accurately calculate the concentration unit of influenza virus HA-specific antibodies, and to normalize the differences between subtype strains, we calculated individual standard curves for each recombinant HA coupled bead set. Using this method, we derived the absolute anti-HA antibody concentration for each influenza strain using a five-parameter logistic regression model. Importantly, we estimated strain-specific parameters for each of the different HA subtype strains in the assay. We then applied this method to data from mPlex-Flu analysis of treatment groups of an influenza vaccine clinical trial (DMID 08-0059 [14]).

The results demonstrated differences in longitudinal estimates and comparisons from linear mixed effects models when comparing different treatment groups. Simulation studies showed that taking HA subtype variations into account in influenza anti-HA multiplex vaccination analysis lowered false discovery rates (FDR) and improved the specificity of the comparisons between treatment groups. Establishing individual standard curves for each influenza virus HA subtype will be extremely useful for the development of broadly cross-reactive influenza vaccines. This method is generalizable to multiplex assays of polyclonal, antibody-mediated immunity against viruses with significant strain variation.

## 2. Methods

### 2.1. Human Subjects Ethics Statement

This study was approved by the Research Subjects Review Board at the University of Rochester Medical Center (RSRB approval number RSRB00012232). The clinical samples were analyzed under secondary use consent, and written informed consent was obtained from all participants and kept on file per RSRB regulations. Research data were coded such that subjects could not be identified, directly or through linked identifiers, in compliance with the Department of Health and Human Services Regulations for the Protection of Human Subjects (45 CFR 46.101(b) [14]. Subject identification numbers were reencoded for publication.

### 2.2. Vaccine Study Design and Sample Collection

**Figure 2:**
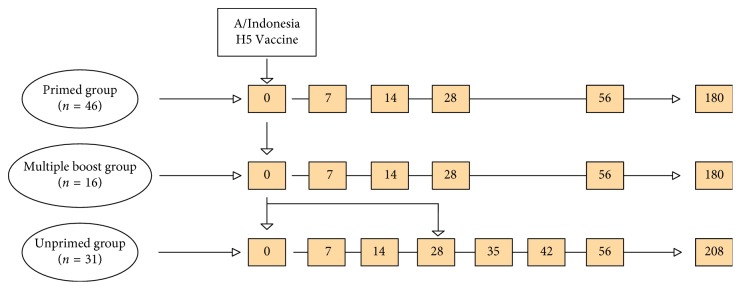
Study design of the prospective clinical trial of H5 influenza vaccination (DMID 08–0059) with an intramuscular inactivated A/Indonesia/5/05 (A/Ind05) H5 influenza vaccine. The inactivated A/Indonesia/5/05 (A/Ind05) intramuscular influenza vaccine was used to vaccinate all subjects in the three cohorts. The inactivated subvirion influenza A/Vietnam/1203/04 (A/Vie04) vaccine in 2005-2006 was used to vaccinate the primed and the multiple boost groups. The baculovirus expressed recombinant influenza A/Hong Kong/156/97 vaccine (A/HK97) in 1997-1998 was also used to vaccinate the multiple primed group. The unprimed group was vaccinated the A/Ind05 vaccine and a second booster vaccine at 28 days. Blood samples were collected as shown in the orange blocks: before vaccination (Day 0) and on days 7, 14, 28, 56, and 180 after vaccination. For the unprimed group, blood samples were collected before vaccination (Day 0) and on days 7, 14, and 28 before boosting on day 28 and then on days 7 (Day 35), 14 (Day 42), 28 (Day 56), and 180 (Day 208) after boosting.

Data and serum samples used in this report were obtained from stored samples generated by a prospective clinical trial of H5 influenza vaccination (DMID 08-0059) [14]. Briefly, 64 previously H5 influenza-vaccinated (PR) and 30 healthy adults not previously vaccinated against H5 influenza strains (UP) were vaccinated with an intramuscular inactivated A/Indonesia/5/05 (A/Ind05) vaccine in two doses (Figure 2). The antibody concentration data from the different doses within the same vaccine treatment group were adjusted in the statistical analysis using linear mixed effects models [15]. All of the PR group received the intramuscular inactivated A/Vietnam/1203/04 (A/Vie04) vaccine in 2005-2006. Of these subjects, 16 had received a vaccine containing the rHA of A/Hong Kong/156/97(A/HK97) in 1997-1998 and are designated as the multiply primed group (MPR). Subjects of PR and MPR groups were administrated single doses of the A/Ind05 influenza vaccine. Subjects in MPR group received 2 identical vaccinations separated by 28 days. Serum samples were collected before vaccination (Day0) and on days 3, 7, 14, 28, 56, and 180 after vaccination. Serum samples were also collected from the UPR group on days 3, 7, 14, and 28 days after the second immunization.

### 2.3. mPlex-Flu Analysis

We estimated concentrations of IgG antibodies against 45 HA strains of influenza viruses in the serum samples from the DMID 08–0059 vaccine study using the mPlex-Flu assay [3]. All recombinant HA (rHA) proteins were produced by our lab, with trimerization domain on the end of C-terminal, including the HA heads. All rHAs were in trimer structure. Briefly, a panel of rHAs coupled mPlex-Flu beads listed on Table 1 were mixed and incubated with 20 *µ*l of diluted human sera for 2 hours, at 500 beads per each bead's region in the 96-well filtration plates (Millipore, Billerica, MA) at 4°C, on a rotary shaker (500 rpm) in the dark. The wells were washed twice and then incubated with 1 : 400 diluted PE conjugated anti-human IgG (*γ* chain specific) secondary antibodies (SouthernBiotech, AL) in the dark at room temperature for 2 hours with gentle agitation (500 RPM). After three additional washes, the beads in each well were suspended in Luminex Magpix Drive Fluid (Luminex, Austin, TX) and analyzed on a MagPix multiplex reader (Luminex, Austin, TX), and the results obtained were measured in median fluorescence intensity (MFI).

### 2.4. Standard Serum and Standard Curves of mPlex-Flu Assay

Positive control serum for the seasonal influenza virus mPlex-Flu (STD02) was created by pooling four positive sera from the subjects who had confirmed high concentrations of IgG antibodies against all H1, H3 seasonal influenza virus strains and most H5 avian influenza virus strains. The total IgG concentration of this serum was 9.07 mg/ml, as estimated by ELISA using a purified IgG standard (Abcam INC, MA, USA). Using this control serum, the traditional standard curve of total IgG concentrations was found using a goat-anti-human IgG Fc specific capture antibody (Sigma-Aldrich, MO, USA) coupled to Luminex beads. We assayed serial dilutions of STD02 serum to create the total IgG standard curve, beginning with an initial dilution of 1 : 1000, followed by serial four-fold dilutions and a blank control [3]. STD02 serum at the same dilutions was then used to generate individual standard curves for each of the 45 influenza strain subtypes [3].

### 2.5. Dose-Response Curve

The MFI-IgG concentration relationship was modeled using four-parameter and five-parameter logistic regression models [16, 17]. For the mPlex-Flu assay, we assume *y*_*is*_ is the response corresponding to dilution level *x*_*i*_ for *s*th the strain subtype. Then *y*_*is*_ and *x*_*i*_ are described by the nonlinear function

(1) $$\begin{array}{c} y_{is}=f\left(x_{i},\theta_{s}\right)+\epsilon_{is}, \end{array}$$

where *ϵ*_*is*_ follows normal distribution with mean *μ* *=* 0 and variance *σ* ^2^ *g*{*f*(*x*_*i*_, *θ*_*s*_), *τ*} where *g*{*f*(*x*_*i*_, *θ*_*s*_), *τ*} is a function of *f*(*x*_*i*_, *θ*_*s*_). The functions *f*(*x*_*i*_, *θ*_*s*_) are different for four-parameter logistic regression models and five-parameter logistic regression models. The four-parameter logistic regression model *f*(*x*_*i*_, *θ*_*s*_) is given by

(2) $$\begin{array}{c} f\left(x_{i},\theta_{s}\right)=\theta_{2s}+\frac{\theta_{3s}-\theta_{2s}}{1+{\left(x_{i}/\theta_{4s}\right)}^{\theta_{1s}}}, \end{array}$$

while the five-parameter model *f*(*x*_*i*_, *θ*_*s*_) is

(3) $$\begin{array}{c} f\left(x_{i},\theta_{s}\right)=\theta_{2s}+\frac{\theta_{3s}-\theta_{2s}}{{\left(1+{\left(x_{i}/\theta_{4s}\right)}^{\theta_{1s}}\right)}^{\theta_{5s}}}, \end{array}$$

where parameters for the *s* ^*th*^ strain subtype are denoted by *θ*_2*s*_ for the minimum and *θ*_3*s*_ for the maximum responses, *θ*_4*s*_ is the concentration that results in 50% response, *θ*_1*s*_ is the relative slope at the 50% response, and *θ*_5*s*_ denotes the asymmetry in the dose-response relationship. When we use a common standard curve for all strain subtypes, the four-parameter and five-parameter logistic regression models will be given by

(4) $$\begin{array}{c} f\left(x_{i},\theta \right)=\theta_{2}+\frac{\theta_{3}-\theta_{2}}{1+{\left(x_{i}/\theta_{4}\right)}^{\theta_{1}}},\\ f\left(x_{i},\theta \right)=\theta_{2}+\frac{\theta_{3}-\theta_{2}}{{\left(1+{\left(x_{i}/\theta_{4}\right)}^{\theta_{1}}\right)}^{\theta_{5}}}, \end{array}$$

where all *θ*s will take the same value for all strain subtypes. Previous studies have found that the five-parameter logistic regression model is superior to the four-parameter model with respect to the accuracy of concentration estimates [18]. Thus, in our simulation studies, we used five-parameter logistic regression model to estimate the mean concentration.

## 3. Results

### 3.1. Standard Curve Generation for Each Strain Subtype

Traditional immunoassays (e.g., ELISA) generally fit one common five-parameter logistic regression model standard curve to all results of antibody binding to influenza HA strain subtypes. For example, when measuring human immune responses, an anti-human IgG capture antibody is often used to bind serial dilutions of IgG from a solution of known concentration, and a secondary indicator antibody is used to measure the mean fluorescence intensity (MFI) versus the IgG concentration for a standard curve [11, 13]. The principle of assays is shown in Figure 1. However, we have found that using this same procedure to generate a single standard curve is problematic for the multiple assay (mPlex-Flu).

**Figure 3:**
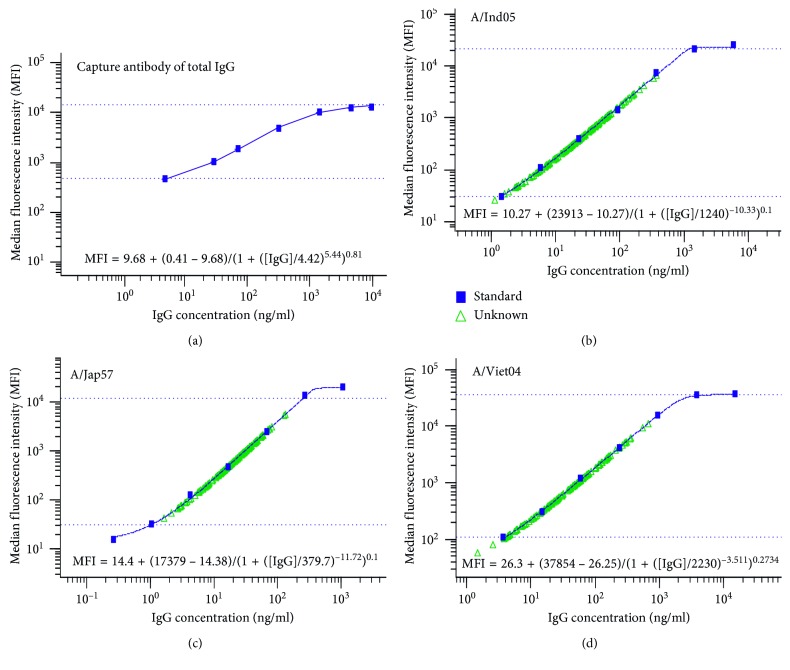
The examples of standard curve and 5PL fitting formula examples generated by mPlex-Flu assay. (a) The standard curves generated with anti-IgG capture antibodies commonly used in traditional immunoassays through fitting a five-parameter logistic regression model. (b–d) The representative subtype-specific standard curves of influenza HAs from total 45 strains, A/Indonesia/5/2005 (A/Ind05, H5), A/Japan/305/1957 (A/Jap57, H2), and A/Viet Nam/1203/2004 (A/Viet04, H5), generated using five-parameter logistic regression models with different parameters for different subtypes. For each graph, the blue markers and line represent a standard curve, either a single curve for multiple strains (a) or a single strain-specific curve (b–d).

We found that the traditional standard curve (Figure 3(a)) generated from the IgG capture antibodies is different from the individual standard curves (Figures 3(b)–3(d) show three influenza strains as examples) generated from each specific influenza HA strain in mPlex-Flu assay, shown as different parameters in the fitted five-parameter logistic regression models. The traditional standard curve has parameters of (*θ*_1_=5.44, *θ*_2_=9.68, *θ*_3_=0.41, *θ*_4_=4.42, *θ*_5_=0.81) in the fitted five-parameter logistic regression model (Figure 3(a)).

The multiple assay approach allowed us to fit a five-parameter logistic regression model for each strain subtype with different parameters for each strain subtypes. For the three strain subtype examples, the estimated parameters in the fitted five-parameter logistic regression models were different as shown in Table 2 and Figures 3(b)–3(d). Table 2 also shows the SEM of those estimated parameters for the three strain subtypes. Therefore, we hypothesize that influenza strain subtype variations (e.g., sequence, density on multiplex bead surface, glycosylation, and charge) could account for or cause inaccuracy during subsequent immunoassay data analysis when comparing different treatment groups in clinical vaccine studies. In addition, with the influenza strain subtype variations being taken into account, it is likely that variations between the strains could be mathematically adjusted for at same time. The data after such adjustment should allow us compare the absolute concentration of IgG anti-influenza between subtype strains. Besides statistical methodology, one can also prepare Ag-specific pools for each of the antigen. This can be done by affinity purification or calibration-free concentration analysis on a BiaCore SPR machine. This is a critical technical problem for the assessment of influenza antibody cross-reactivity in most antibody response and vaccine studies.

### 3.2. Differences in Statistical Inferences with and without considering Strain Subtype Variations

In order to test our hypothesis, we compared the differences in antibody concentrations across three different vaccine treatment groups with data generated by mPlex-Flu assay. First, we used the linear mixed effects model to adjust IgG titres to 21 strains of H5 influenza viruses with the effects of age at enrollment, gender, ethnicity, dosages, and batches. Autoregressive 1 correlation structure was used in the linear mixed effects model to take into account the within-subject correlations [19]. Then, we compared the antibody concentration data against 21 strains of H5 influenza viruses using either (1) a traditional common standard curve used for all strain subtypes (Figure 3(a)) (without considering strain differences) or (2) an individual standard curve for all H5 strain subtypes to consider the strains' difference. The longitudinal concentration data in the logarithm were from three immunization treatment groups: unprimed (UP), primed (PR), and multiple primed (MPR). The data included 21 H5 vaccine strain subtypes with 18606 total observations from 3 different groups, 2 different dosages (15 mcg and 90 mcg), 5 different batches, 7 or 10 different days, and 93 different subjects. The concentration in the logarithm was checked to follow an approximately normal distribution; thus, linear mixed effects models were used to fit the log transformed concentration data to compare the three different vaccine groups with and without considering the H5 vaccine strain subtype variations [20, 21].

**Figure 4:**
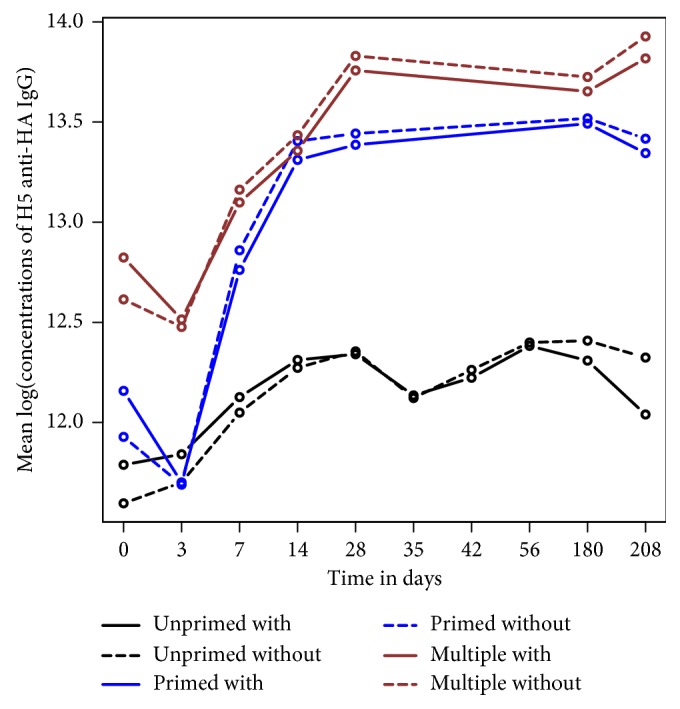
The longitudinal estimates from linear mixed effects models with and without considering the H5 strain subtype variations. The mean of longitudinal log concentration showed a total of 21 strains of H5 anti-HA IgG for each of the three different vaccine treatment groups (UPR, PR, and MPR), with and without strain subtype variation taken into account estimated from the linear mixed effects models with adjustment for the differences in dosages, batches, gender, ethnicity, and time points using the restricted maximum likelihood methods.

For the group comparisons, the Kenward–Roger method was used to estimate the standard error for fixed effects and the degrees of freedom for each parameter [22]. Restricted maximum likelihood estimators were used to obtain the parameter estimates in the linear mixed effects model.  Figure 4 shows the longitudinal estimates of the three different vaccine treatment groups from linear mixed effects models with and without consideration of the H5 strain subtype variations. We noticed that the estimated mean concentrations in the logarithm are different, especially at baseline and end of study measurements. Thus, biases might be introduced to the estimated mean concentrations when strain subtype variations are not taken in account in the data analysis when comparing different treatment groups in clinical vaccine studies. The results shown in Figure 4 suggest that the parameters relating MFI to protein concentration estimated parameters from the five-parameter logistic regression models are different for different H5 strain subtypes and are also different from the estimated parameters in the common standard curve used for all H5 strain subtypes.

**Figure 5:**
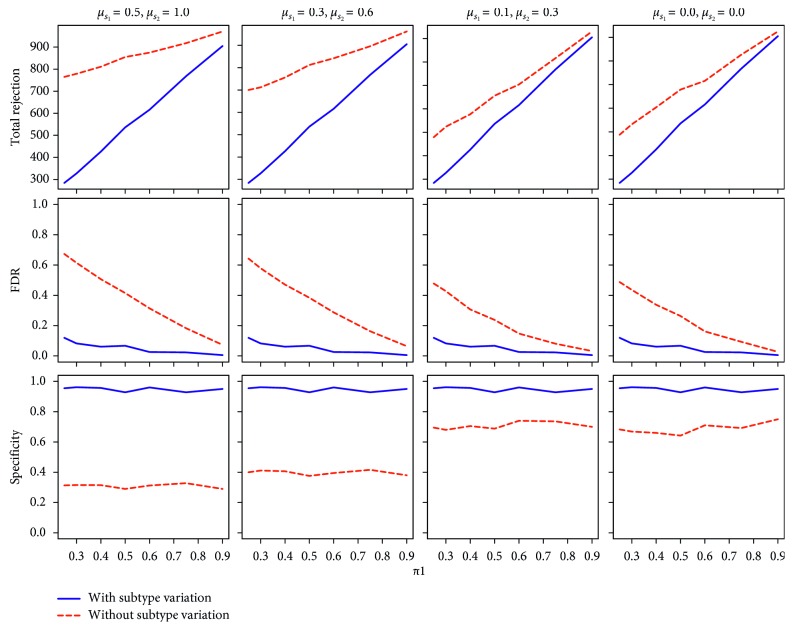
Simulation results on total rejection, FDR, sensitivity, and specificity under different simulation settings with sample size of 15 in each group. The FDRs for comparing different groups were markedly smaller when the strain subtype variations were considered in the data analysis, especially when the proportion of true differences between treatment groups (*π*_1_) were small. The specificities were much larger when the strain subtype differences were taken into account.

Then, we conducted pairwise comparisons between different treatment groups across different time points within the framework of the linear mixed effects model (Figure 5). Among the 276 pairwise comparisons resulting from the linear mixed effects models conducted in SAS v9.4 (SAS Institute Inc., Cary, NC), 234 pairwise comparisons showed significant differences when the strain subtype variation was taken into account. Meanwhile, 230 out of 276 pairwise comparisons showed significant differences when the strain subtype variation was not taken into account in the data analysis. Although there were only 4 differences in the total number of rejections, there were 22 pairwise comparisons having inconsistent results between the analysis taking the strain subtype variation into account and the analysis not taking the strain subtype variation into account.  Table 3 gives some examples of inconsistent results in those pairwise comparisons. It is noticeable from Table 3 that some significant differences might be missed and some significant differences might be false positives if subtype variation is not taken into account in the data analysis. Examples from Table 3 indicate the results of group comparisons in vaccine studies will be affected when strain subtype variation is not taken into account in vaccine data analysis.

### 3.3. Simulation Study

Monte Carlo simulation studies [23] were conducted to assess the importance of including strain subtype variation in immunoassay when comparing different treatment groups. We suspect that both false positives and false negatives will be inflated if strain subtype variations are not taken into account in vaccine data analysis when comparing different treatment groups, given what we have observed in our analysis of the H5 vaccine data (Table 3). Therefore, the FDR, sensitivity, and specificity were compared between group differential analyses with and without considering strain subtype variations.

### 3.4. Simulation Description

Using above mPlex-Flu assay data from H5 clinical trial, we estimated the overall mean concentration from the five-parameter logistic regression model with coefficients estimates of $\hat{\theta }=\left(5.44,9.68,0.41,4.42,0.81\right)$, which gave an overall mean concentration of 12.35 in the simulation. The logarithm of IgG antibody reactivity levels *y*_*ijk*_^*∗*^ from *i*th treatment group, *j*th influenza virus strain subtype, and *k*th sample (*i*=1,2; *j*=1,2,3; *k*=1,2,…, *n*) was simulated according to the following linear regression models:

(5) $$\begin{array}{c} y_{ijk}^{*}=\mu_{0}+g_{i}+s_{j}+\epsilon_{ijk}, \end{array}$$

where *μ*_0_=12.35, *g*_1_ denotes the difference between the first treatment group and the second treatment group that takes sequential values from 1 to 2 for true difference situation and takes a value of 0 for no difference situation, and *g*_2_ is set at 0 in the simulation. The strain subtype difference was denoted by *s*_*j*_. For simplicity, three strain subtypes were included in the simulation. The parameter *s*_1_ denoted the difference between strain subtype 1 and strain subtype 3, which follows a random normal distribution with mean *μ*_*s*1_ and standard deviation of 0.08. Similarly, *s*_2_ denotes the difference between strain subtype 2 and strain subtype 3, which follows a random normal distribution with mean *μ*_*s*2_ and standard deviation *σ*=0.1. The parameter *s*_3_ is set at 0 in the model.

In the simulations, we used four sets of combinations of *μ*_*s*1_ and *μ*_*s*2_ in the simulation studies: (1) *μ*_*s*1_=0.5, *μ*_*s*2_=1.0; (2) *μ*_*s*1_=0.3, *μ*_*s*2_=0.6; (3) *μ*_*s*1_=0.1, *μ*_*s*2_=0.3; and (4) *μ*_*s*1_=0.0, *μ*_*s*2_=0.0. The parameter *ϵ*_*ijk*_ denotes random errors that have independently identical normal distributions with mean *μ*=0 and variance *σ* ^2^ *μ*_0_^*τ*^. According to the experimentally measured IgG concentration data, plausible values for *σ* could be (0.05, 0.08, 0.1) and plausible values for *τ* could be sequential values from 0.5 to 1.2 with an interval of 0.1. We used *μ*_0_=12.35 to obtain a mean variance value of 0.06, i.e., mean{var(*ϵ*_*ijk*_)}=mean{*σ* ^2^ *μ*_0_^*τ*^}=0.06.

We simulated 1,000 random samples of paired data (*y*_*ijk*_^*∗*^, *x*_*ijk*_) from the linear regression equation, where *x*_*ijk*_ is the design matrix for the linear regression model. Among the 1,000 random samples, the proportion with true differences between the treatment groups was set at *π*_1_ (*π*_1_=0.25, 0.30, 0.40, 0.50, 0.60, 0.75, 0.90). When *π*_1_=0.25, there are 1,000 × *π*_1_=1,000 × 0.25=250 random samples having true differences between the treatment groups. The magnitude of the true differences ranged from 1-2 with an increment of ((2 − 1)/250)=0.004 from the first to the 250th random sample. The true difference between the treatment groups is 0 for the remaining 750 samples in the simulation. The sample size for each random sample was set at *n*=15 for each of the treatment groups.

Traditional approaches to quantify the IgG antibodies against different influenza HA subtypes in human serum use a common standard curve to estimate the IgG concentration across all strains and subtypes. Such approaches generally test for a statistically significant difference between treatment groups without considering subtype antibody binding variations. The following regression models are commonly used to fit the logarithm of the concentration data:

(6) $$\begin{array}{c} y_{ik}=\mu_{0}+g_{i}+\epsilon_{ik},\;i=1,2;k=1,\ldots ,n, \end{array}$$

where *y*_*ik*_ denotes the logarithm of the concentration data for *i*th group and *k*th sample within the *i*th group, *g*_1_ denotes the differences between treatment groups and *g*_2_=0. *ϵ*_*ik*_ is assumed to have independent identical distribution of *N*(0, *σ* ^2^).

Our approach takes the variation in subtype antibodies and reagents into account. We fit a standard curve for each of the viral HA subtypes using a five-parameter logistic regression model with strain-specific parameters fitted to the logarithm of the concentration data:

(7) $$\begin{array}{c} y_{ijk}=\mu_{0}+g_{i}+s_{j}+\epsilon_{ijk}, \end{array}$$

where *y*_*ijk*_ denotes the logarithm of the concentration data for *i*th group, *j*th strain, and *k*th sample within the *i*th group *j*th strain, *g*_1_ denotes the differences between treatment groups and *g*_2_=0, *s*_1_ denotes the difference between strain subtypes 1 and 3, *s*_2_ denotes the difference between strain subtypes 2 and 3, and *s*_3_=0. *ϵ*_*ik*_ is assumed to have independent identical distribution of *N*(0, *σ* ^2^). To simplify our simulation studies, we assumed that the correlations among the three subtypes were zero.

### 3.5. Simulation Results: Improved FDR Control and Specificity with Strain Subtype Variation Considered

Figure 5 shows the simulation results comparing the treatment groups with and without strain subtype differences taken into account. We next sought to determine if accounting for influenza HA strain variation would affect the statistical comparison of vaccine response treatment groups from a clinical influenza vaccine study. The data were generated from an mPLEX-flu assay of samples collected during a previous study of responses to an H5 influenza vaccine (DMID 08-0059) [14]. The goal of the trial was to determine if there were significant differences in the anti-HA influenza antibody response between three groups that received intramuscular anti-A/Indonesia/5/05 H5 influenza vaccine: UPR—no previous exposure to any H5 vaccine, PR—had been vaccinated once previously against a different H5 influenza strain (either A/Vietnam/1203/04 or a recombinant HA vaccine against A/Hong Kong/156/97(A/HK97)), and MPR—received two sequential vaccinations against the A/Indonesia/5/05 H5 influenza virus.

The FDRs for comparisons between different treatment groups were markedly smaller and the specificity much greater, when the strain subtype differences were taken into account. When differences in strain subtype were not accounted for, the probability of finding differences between treatment groups was much higher with more significant differences identified between the treatment groups. In contrast, considering strain subtype variation did not affect the sensitivity of statistical comparison between different treatment groups.

We also observed a noticeable decrease in FDRs as the proportion of true differences between the treatment groups increased. In contrast, the differences in specificities did not change even as the proportion of true differences between the treatment groups increased. When the variation in influenza HA subtype differences decreased, we found a concomitant decrease in FDRs and an increase in specificity (Table 4). Even when the mean concentration difference between strain subtypes was zero, the FDRs were still much larger and the specificities were still much smaller when comparing differences between the treatment groups. The inflated FDRs appeared largely due to ignoring strain subtype variations and increased as the proportion of true differences between the treatment groups became smaller.

When the sample size in the simulation studies increased to 30, 60, and 120 in each treatment group, we obtained similar results for the number of total rejections, FDR, sensitivity, and specificity (Supplementary Figures 1–3). Thus, only the results from sample size of 15 in each treatment group are presented in Figure 5.

## 4. Discussion

Every year, the WHO selects influenza vaccine strains trying to pick the best influenza virus strains that would be able to represent the circulating virus strains in same or similar antigenicity of HA protein, on the surface of virus. Furthermore, the traditional way for determination of the antigenicity of one influenza virus is to let this virus isolation to react against a panel of ferret antisera, and each antiserum is generated from naive ferret after the infection of one single-specific influenza virus [2]. However, antigenic data or immunological patterns in human sera are more complicated and difficult to interpret due to exposure histories and cross-reactivity between influenza virus strains [1]. In addition, some important studies showed that early lifetime exposure of influenza virus (imprinting) might provide cross-protection against infection of H5, H7 novel subtype influenza viruses [24]. It is essential to develop an efficient and high throughput assay for the evaluation of those cross-reactive antibodies. This novel technique, mPlex-Flu assay, allows us be able to quickly and accurately estimate the humoral immune response after influenza infection or vaccination and rapidly characterize comprehensive individual- and population-level heterosubtypic immunity of a broad range of influenza strains.

However, how to quantify the antibodies and their cross-reactivities against influenza virus is always a challenge in influenza vaccine studies. The mFlex-Flu assay couples beads with analyte-specific rHAs (antigens) to detect the specific antibodies binding influenza strain-specific HAs. To quantify the amount of active HA-specific antibodies, mPlex-Flu assay assesses both the amount and affinity of antibodies against influenza viral HAs at the same time. Furthermore, there are many modest differences between the replication and infection of subtypes of influenza viruses [25] and the diversity of HA structure and characters between individual subtypes of influenza viruses [26]. In addition, the slight differences in batch, time, and other experiment conditions can also introduce variation from assay to assay. Traditional assays to evaluate specific antibodies against influenza subtype virus or HA, such as HAI [27], MN [28], and ELISA [29], are semiquantitative. They use the highest dilution or endpoint of dilution of serum to determine the titer of the antibodies. Those discrete-ranked readouts of one of 8–14 titer values could introduce imprecision and increase false discoveries. The major problem is that they are not able to provide a precise evaluation normalized by the difference in strains of influenza virus. This presents a big challenge for directly comparing the anti-HA IgG levels against different influenza viruses, within or between subtype of influenza virus, when studying the frequency and binding of cross-reactive antibodies against multiple influenza strains.

In order to generate precise continuous values of antibody levels, which rely on the appropriate standard sample to generate a good standard curve, initially, we set up an ELISA assay with the anti-human IgG antibody standard curve, as described previously [11, 13]. Although using one standard curve can adjust for variation caused by experiment conditions, we still could not determine the types of imprecisions introduced by the variations of deferent strains HAs of influenza virus. In our previous publication [3, 4], we introduced a novel multiplex method to quantitatively measure the concentration of rHA-specific antibodies by using the standard reference serum (STD02), similar to SDT01 [3], which is a mixture serum from four subjects having high titer antibodies against seasonal influenza viruses, based on a study of the estimation of weight-based antibody unite, published by Dr. Quataert [12]. Importantly, we also set up independent standard curves for each analyte (influenza virus HA strain) to convert the MFI units of mPlex-Flu assay into concentration-based antibody units.

Within the mPLEX-Flu assay, the HA from various strains do not directly interact. The one class of interactions that could influence the assay is that of competition for antibodies that bind to regions of different HA strains having the same antigenic sequence (epitopes). In our experimental setting, we use excess serum (with antibodies to multiple antigens) or monocloncal antibodies (bind to a single antigenic site) to minimize or eliminate the binding competition between influenza virus strains. Under these conditions, the binding of anti-HA IgG to one HA variation does not affect binding of another HA variation given there are more than enough antibodies available. Similarly, the standard curve of each subtype antibody concentration can be generated to obtain the antibody concentration for each HA subtype. Thus, the binding of each HA variation with antibodies can be treated as independent binding. We are aware that these conditions may not hold true at much lower antibody concentrations but have found such concentrations to be below the usual range for serum antibody. However, it is important to note that the independence assumption gives us more conservative results for our statistical evaluation than dependent assumption. Thus, the simulation results we obtained using the independent assumption are still valid for dependent situation.

The results of this study showed significant variations in IgG-rHA binding model parameter estimates among different rHA strains. This finding is likely due to differences in reagent surface density and staeric hinderence between subtypic recombinant HA proteins (rHAs). When different standard curves were used for each strain subtype, the concentration differences between different strain subtypes could be taken into account when comparing different treatment groups. When one common standard curve was used for all strain subtypes, the concentration differences between different strain subtypes were embedded in the random errors.

Our simulation studies have shown that without considering the variation in strain subtypes, the Type I error associated with testing differences between treatment groups will be inflated and the specificity will be lowered, compared to analysis with the strain subtype variation taken into account. Our case studies also showed inconsistent results in pairwise group comparisons when we took or did not take the variations in strain subtypes into account. Therefore, the estimated differences of interested group comparisons are less biased if the strain subtype variations are taken into account in the data analysis by estimating concentrations from the individual standard curves of each strain subtype. Meanwhile, the type I error of testing interested group differences will be reduced and the specificity will also be increased. Thus, we recommend taking strain subtype variations into account in clinical vaccine research.

This study provides solid statistic evidence to support our published method to quantify the concentration unit of antibodies in mPlex-Flu assay. It also suggests that it is more accurate to directly compare the concentration units between subtype analytes after adjustment by each individual standard curve.

## Figures and Tables

**Table 1:** The mPlex-Flu assay panel of seasonal influenza viruses, H5 clades, and subclades.

Influenza virus type	Subtypes	Full name of viruses	Abbreviation	H5 clades/subclades
A	H1	A/South Carolina/1/18	A/SC18	
		A/Puerto Rico/8/1934	A/PR8	
		A/USSR/90/1977	A/USSR77	
		A/New Caledonia/20/1999	A/NewCall99	
		A/Texas/36/1991	A/Tex91	
		A/California/07/2009	A/Cali09	
	H2	A/Japan/305/1957	A/Jap57	
	H3	A/Port Chalmers/1/1973	A/PC73	
		A/Hong Kong/1/1968	A/HK68	
		A/Perth/16/2009	A/Per09	
		A/Victoria/361/2011	A/Vic11	
		A/Texas/50/2012	A/Tex12	
	H5	A/Hong Kong/156/97	A/HK97	0
		A/Viet Nam/1203/2004	A/Viet04	1
		A/Cambodia/P0322095/2005	A/Cam05	1.1
		A/Indonesia/5/05	A/Ind05	2.1.3.2
		A/Turkey/65596/2006	A/TK06	2.2.1
		A/Common Magpie/Hong Kong/5052/2007	A/cmHK07	2.3.2.1
		A/Shenzhen/406H/2006	A/SZ06	2.3.4
		A/Chicken/Guangxi/12/2004	A/chiGX04	2.4
		A/Chicken/Korea/es/2003	A/chiKR03	2.5
		A/Silky Chicken/Hong Kong/SF189/01	A/s.chiHK01	3
		A/Goose/Guiyang/337/2006	A/gooGY06	4
		A/Duck/Guangxi/1378/2004	A/ducGX04	5
		A/Duck/Hubei/wg/2002	A/ducHB02	6
		A/Beijing/01/2003	A/BJ03	7.1
		A/Chicken/Shanxi/2/2006	A/chiSX06	7.2
		A/Chicken/Henan/16/2004	A/chiHN04	8
		A/Goose/Shantou/1621/05	A/gooST05	9
		A/duck/Sichuan/NCXN10/2014(H5N1)	A/ducSC14	2.3.4.4
		A/turkey/Washington/61–22/2014(H5N2)	A/turWash14	2.3.4.4
		A/duck/Guangdong/wy11/2008(H5N5)	A/ducGD08	2.3.4.4
		A/turkey/California/K1500169-1.2/2015(H5N8)	A/turCal15	2.3.4.4
	H6	A/chicken/Taiwan/67/2013	A/chTW13	
	H7	A/mallard/Netherlands/12/2000	A/malNert00	
		A/rhea/North Carolina/39482/1993	A/rheaNC93	
	H9	A/guinea fowl/Hong Kong/WF10/1999	A/gfHK99	

B		B/Brisbane/60/2008	B/Bris08	

HA domains		Head of A/Shanghai/1/2013	H7 Head	
		Head of A/Indonesia/5/05	H5 Head	
		Headof A/guinea fowl/Hong Kong/WF10/1999	H9 head	

Chimeric HA		cH5/1 (head of A/Ind05, stalk of A/PR8)	cH5/1PR	
		cH5/1 (head of A/Ind05, stalk of A/Cal09)	cH5/1Cal	
		cH9/1 (head of A/gf/HK99, head of A/Cal09)	cH9/1	
		cH4/7 (Head of A/duck/Czech/1956, stalk of A/Shanghai/1/2013)	cH4/7	

*Note*. All antigens were trimeric.

**Table 2:** Example of estimated parameter variations in three HA strain subtypes.

HA strain subtype	Estimated parameters				
	*θ*_1_	*θ*_2_	*θ*_3_	*θ*_4_	*θ*_5_
A/Indonesia/5/2005	−10.33	10.27	23913	1240	0.1
A/Japan/305/1957	−11.72	14.4	17379	379.7	0.1
A/Viet Nam/1203/2004	−3.511	26.25	37854	2230	0.2734
SEM of estimated parameters	2.54	4.79	6038.17	534.57	0.058

SEM = standard error of the mean.

**Table 3:** Examples of inconsistent results from pairwise comparisons with and without consideration of strain subtype variations in the clinical vaccine data analysis.

Group	Time in days	Group	Time in days	With consideration of subtype variation			Without consideration of subtype variation		
				Δ	SE of Δ	*P* value	Δ	SE of Δ	*P* value
MPR	0	MPR	3	0.3092	0.0643	<0.0001	0.1376	0.0825	0.0953
MPR	180	PR	180	0.1609	0.0956	0.0927	0.2062	0.0938	0.0281
PR	14	PR	180	−0.1812	0.0494	0.0002	−0.1139	0.0610	0.0620
PR	28	PR	180	−0.1056	0.0453	0.0198	−0.0762	0.0583	0.1913
UP	56	UP	208	0.3424	0.0842	<0.0001	0.0751	0.1031	0.4663
UP	180	UP	208	0.2689	0.0666	<0.0001	0.0843	0.0854	0.3237

SE = standard error; PR = primed group; MPR = multiply primed group; UP = unprimed group.

**Table 4:** FDR and specificity comparison with and without considering the strain subtype variations in the treatment groups comparisons at different settings.

Parameter settings	*π*_1_	With	Without	With	Without
		FDR		Specificity	
	0.25	0.1197	0.6732	0.9547	0.3133
	0.30	0.0826	0.6149	0.9614	0.3157
Setting 1	0.40	0.0610	0.5068	0.9567	0.3150
*μ*_*s*1_=0.5	0.50	0.0672	0.4152	0.9280	0.2900
*μ*_*s*2_=1.0	0.60	0.0260	0.3143	0.9600	0.3125
	0.75	0.0234	0.1830	0.9280	0.3280
	0.90	0.0055	0.0731	0.9500	0.2900

	0.40	0.0610	0.4709	0.9567	0.4067
	0.50	0.0672	0.3842	0.9280	0.3760
Setting 2	0.60	0.0260	0.2874	0.9600	0.3950
*μ*_*s*1_=0.3	0.75	0.0234	0.1629	0.9280	0.4160
*μ*_*s*2_=0.6	0.90	0.0055	0.0644	0.9500	0.3800
	0.25	0.1197	0.4781	0.9547	0.6947
	0.30	0.0826	0.4275	0.9614	0.6800

	0.40	0.0610	0.3068	0.9567	0.7050
	0.50	0.0672	0.2378	0.9280	0.6880
Setting 3	0.60	0.0260	0.1477	0.9600	0.7400
*μ*_*s*2_=0.1	0.75	0.0234	0.0809	0.9280	0.7360
*μ*_*s*2_=0.3	0.90	0.0055	0.0323	0.9500	0.7000
	0.25	0.1197	0.4877	0.9547	0.6827
	0.30	0.0826	0.4361	0.9614	0.6686

	0.40	0.0610	0.3377	0.9567	0.6600
Setting 4	0.50	0.0672	0.2636	0.9280	0.6420
*μ*_*s*1_=0.0	0.60	0.0260	0.1620	0.9600	0.7100
*μ*_*s*2_=0.0	0.75	0.0234	0.0931	0.9280	0.6920
	0.90	0.0055	0.0270	0.9500	0.7500

FDR = false discovery rate.

## Data Availability

The data used to support the findings of this study are available from the corresponding author upon request.

## Supplementary Materials

Supplementary Materials Supplementary Figures 1–3: simulation study results with total rejections, FDR, sensitivity, and specificity when the sample size is 30, 60, or 120 in each treatment group.
