# Supplementary material for: Improved Specificity and False Discovery Rates for Multiplex Analysis of Changes in Strain-Specific Anti-Influenza IgG
Source: Comput Math Methods Med. 2019 Apr 15;2019:3053869. doi: 10.1155/2019/3053869 (PMC6501432; doi:10.1155/2019/3053869)
Supplement: Supplementary Materials — Supplementary Figures 1–3: simulation study results with total rejections, FDR, sensitivity, and specificity when the sample size is 30, 60, or 120 in each treatment group. [file 3053869.f1.zip › Influenza_strain_calculation_comparison_supplemental_document.pdf]

SUPPLEMENTAL MATERIALS

Title: Improved Specificity and False Discovery Rates for Multiplex Analysis of Changes in Strain Specific Anti-Influenza IgG

Supplementary Figure 1 - 3: The simulation study results with total rejections, FDR, sensitivity, and specificity When the sample size is 30, 60, or 120 in each treatment group.

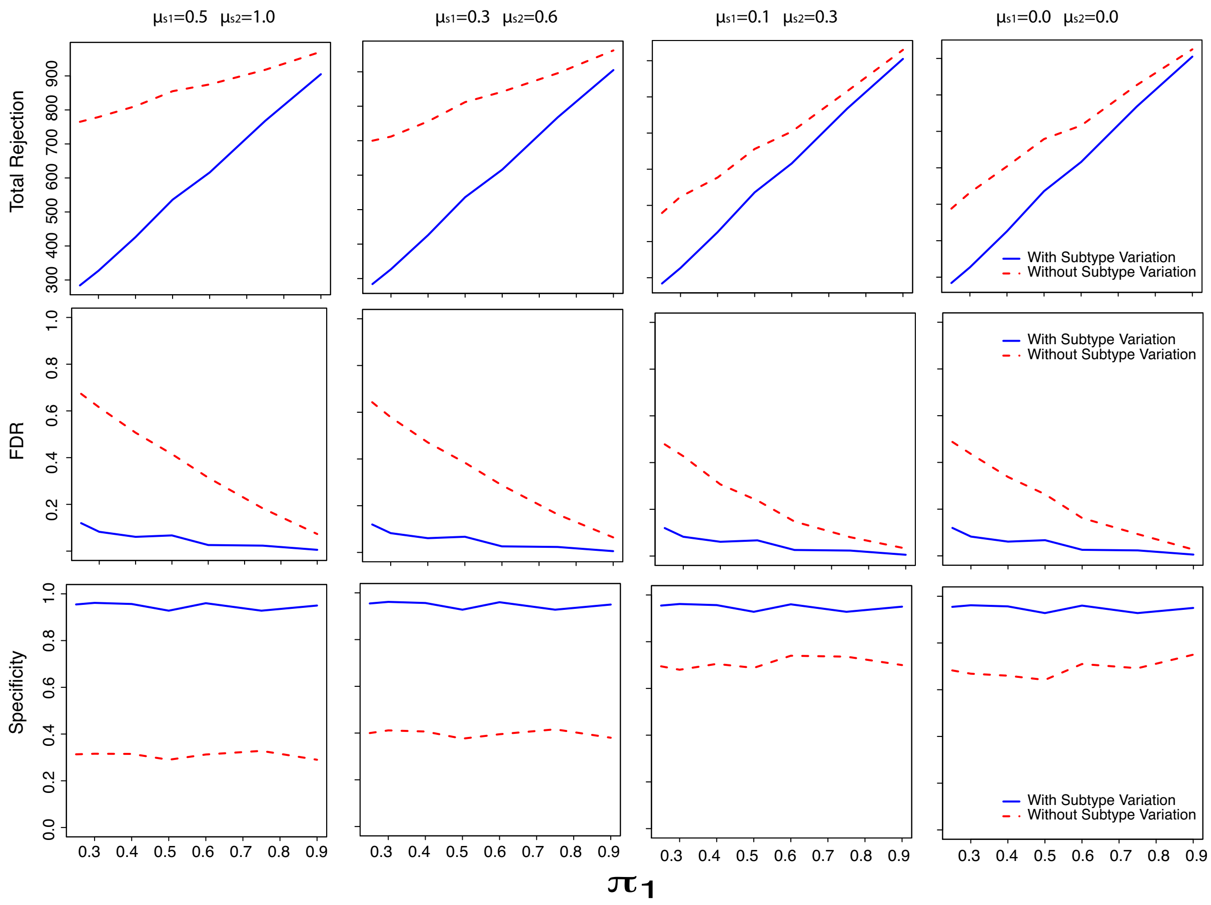

Figure 1: Simulation results on total rejection, FDR, sensitivity, and specificity under different simulation settings with sample size of 30 in each group.

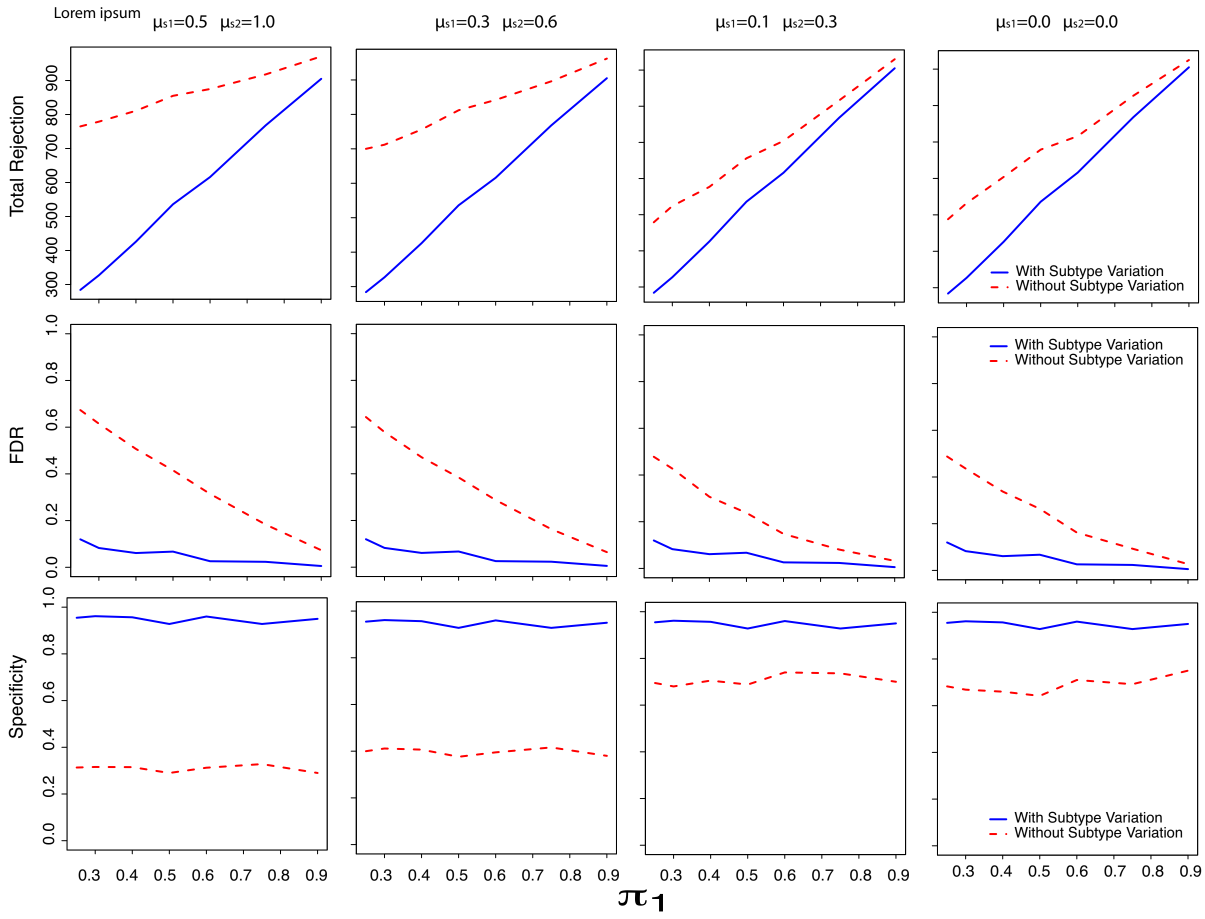

Figure 2: Simulation results on total rejection, FDR, sensitivity, and specificity under different simulation settings with sample size of 30 in each group. The FDRs for comparing different groups were markedly smaller when the strain subtype variations were considered in the data analysis, especially when the proportion of true differences between treatment groups (π_1) were small. The specificities were much larger when the strain subtype diffrences were taken into account.

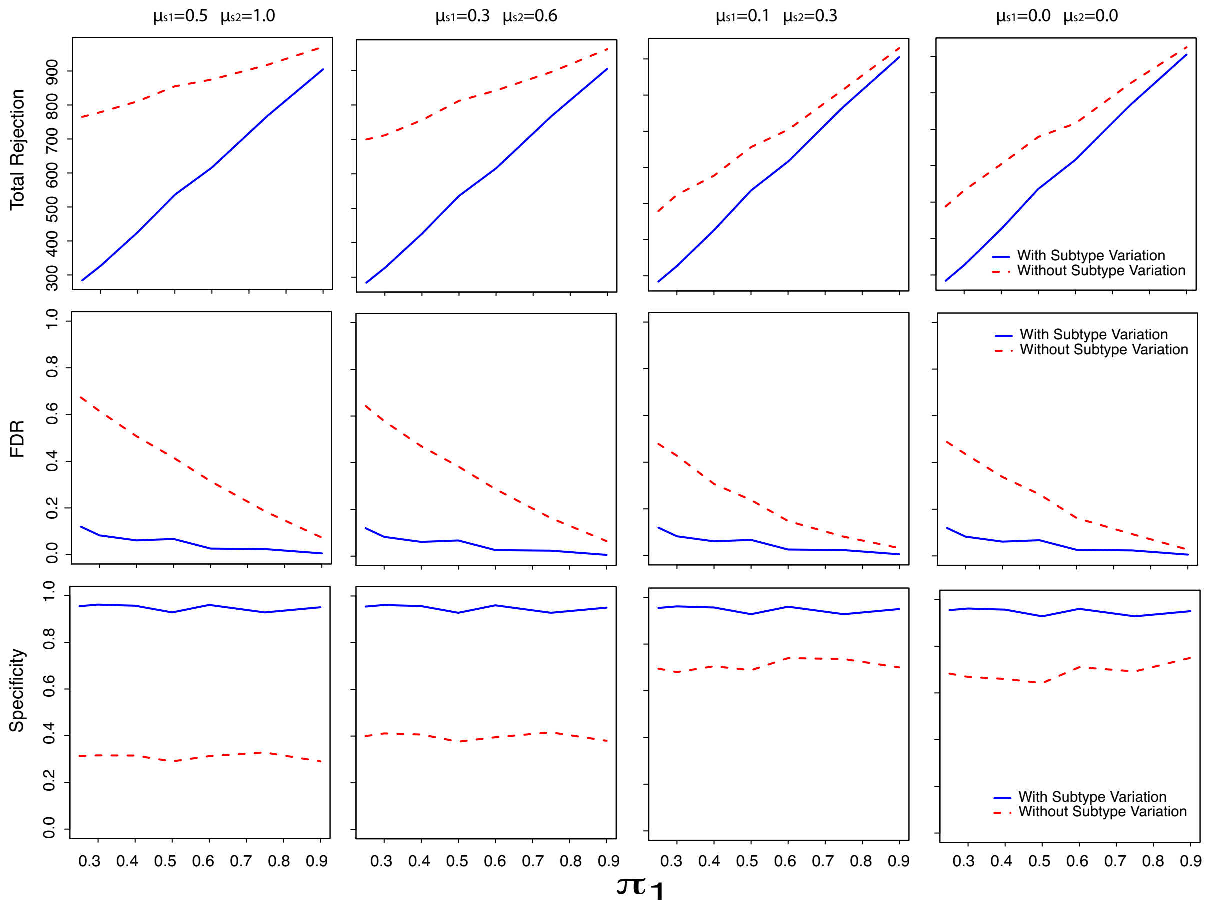

Figure 3: Simulation results on total rejection, FDR, sensitivity, and specificity under different simulation settings with sample size of 30 in each group. The FDRs for comparing different groups were markedly smaller when the strain subtype variations were considered in the data analysis, especially when the proportion of true differences between treatment groups (π_1) were small. The specificities were much larger when the strain subtype diffrences were taken into account.
